# Artificial Intelligence in the Differential Diagnosis of Cardiomyopathy Phenotypes

**DOI:** 10.3390/diagnostics14020156

**Published:** 2024-01-10

**Authors:** Riccardo Cau, Francesco Pisu, Jasjit S. Suri, Roberta Montisci, Marco Gatti, Lorenzo Mannelli, Xiangyang Gong, Luca Saba

**Affiliations:** 1 Department of Radiology, Azienda Ospedaliero Universitaria (A.O.U.), di Cagliari-Polo di Monserrato s.s. 554 Monserrato, 09045 Cagliari, Italy; riccardocau00@gmail.com (R.C.); fra.pisu1@gmail.com (F.P.); 2 Stroke Monitoring and Diagnostic Division, AtheroPoin™, Roseville, CA 95661, USA; jsuri@comcast.net; 3 Department of Cardiology, Azienda Ospedaliero Universitaria (A.O.U.), di Cagliari-Polo di Monserrato s.s. 554 Monserrato, 09045 Cagliari, Italy; rmontisci@unica.it; 4 Department of Radiology, Università degli Studi di Torino, 10129 Turin, Italy; m.gatti@unito.it; 5 IRCCS SynLab SDN S.p.A., 80143 Naples, Italy; mannellilorenzo@yahoo.it; 6 Radiology Department, Zhejiang Provincial People’s Hospital, Affiliated People’s Hospital, Hangzhou Medical College, Hangzhou 310014, China; gong.xy@vip.163.com

**Keywords:** artificial intelligence, deep learning, machine learning, cardiomyopathy

## Abstract

Artificial intelligence (AI) is rapidly being applied to the medical field, especially in the cardiovascular domain. AI approaches have demonstrated their applicability in the detection, diagnosis, and management of several cardiovascular diseases, enhancing disease stratification and typing. Cardiomyopathies are a leading cause of heart failure and life-threatening ventricular arrhythmias. Identifying the etiologies is fundamental for the management and diagnostic pathway of these heart muscle diseases, requiring the integration of various data, including personal and family history, clinical examination, electrocardiography, and laboratory investigations, as well as multimodality imaging, making the clinical diagnosis challenging. In this scenario, AI has demonstrated its capability to capture subtle connections from a multitude of multiparametric datasets, enabling the discovery of hidden relationships in data and handling more complex tasks than traditional methods. This review aims to present a comprehensive overview of the main concepts related to AI and its subset. Additionally, we review the existing literature on AI-based models in the differential diagnosis of cardiomyopathy phenotypes, and we finally examine the advantages and limitations of these AI approaches.

## 1. Introduction

Cardiomyopathy is defined as abnormal myocardial structure and function in the absence of coronary artery disease, hypertension, valvular disease, or congenital heart diseases [1]. According to the recent European Society of Cardiology (ESC) guidelines, cardiomyopathies are now divided into five major subtypes, namely: hypertrophic cardiomyopathy (HCM), dilated cardiomyopathy (DCM), arrhythmogenic right ventricular cardiomyopathy (ARVC), non-dilated left ventricular cardiomyopathy (NDLVC), and restrictive cardiomyopathy (RCM) [1].

Early detection, diagnosis, and treatment are significant in slowing the progression of cardiomyopathy to advanced diseases and improving overall outcomes, not only for the individual patient, but also for the family as a whole [1,2].

Patients with cardiomyopathy may access health services through a heterogeneous clinical presentation or incidental findings, such as electrocardiogram abnormalities during routine sports visits or family screening [1,2,3]. A multidisciplinary approach is required in order to evaluate this complex cardiac condition, aiming to establish and characterize the cardiomyopathy phenotypes and the underlying etiological diagnosis [3]. This phenotype-based etiological diagnosis necessitates the integration of various data, including personal and family history, clinical examination, electrocardiography, and laboratory investigations, as well as multimodality imaging [1]. 

Novel approaches based on artificial intelligence (AI), and on the subsets of machine learning (ML) and deep learning (DL) [4,5,6,7,8], could provide more accurate stratification and typing for patients with cardiomyopathy, presenting a potential solution to overcome the limitations associated with traditional approaches and optimize personalized medicine [9]. Indeed, one of the many applications of AI and its subsets is to create models that consider a large amount of data, including laboratory information and clinical, molecular, and imaging data, for the superior diagnostic and prognostic stratification of patients. Developing effective knowledge systems that integrate different layers of data is critical to maximizing the impact on translational research and personalized medicine. Currently, there is limited research that directly investigates the integration of these algorithms into clinical practice and verifies their positive impact on clinical outcomes. Additionally, few reviews have analyzed the impact of AI in the classification of cardiomyopathies, and, to the best of our knowledge, none have focused on the impact of AI models in the differential diagnosis of cardiomyopathy phenotypes.

The purpose of this review is to provide an overview of AI models developed to discriminate between cardiomyopathy phenotypes, following the recent ESC guidelines for the management of cardiomyopathies, and to highlight the benefits of their application in clinical practice. Finally, we discuss the current limitations of these AI models and their potential future development.

## 2. Notion of AI

From the invention of the term “artificial intelligence” in 1956 by John McCarthy, the research and development of AI have not stopped. AI is a broad term encompassing a variety of methods that enable machines to gain knowledge through experience and replicate human cognitive functions [6,10,11,12,13]. Several subfields of AI exist, namely, machine learning (ML) and deep learning (DL). Table 1 summarizes an overview of AI models with a brief description.

### 2.1. Machine Learning

ML refers to the ability of AI systems to extract patterns in raw data without being explicitly programmed to do so. It involves training a model through the utilization of data with known ground-truths to subsequently generate predictions for new, unseen inputs [12,14]. ML models can be broadly classified into different classes according to the type of experience which they are authorized to undergo throughout their training processes, including supervised, unsupervised, and hybrid paradigms such as semi-supervised learning, weakly supervised learning, and self-supervised learning [7,15]. Supervised learning requires annotated data, also known as “labels”. These labeled data are usually evaluated by an expert physician and represent the ground truth data used in the training process. The algorithm involves iteratively adjusting to minimize the disparity between predicted outputs and actual labels, with the aim of understanding the relationship between input data and output labels. The inclusion of expert evaluation and annotation makes these models more time-consuming and resource-intensive compared to unsupervised and hybrid paradigms in machine learning. Examples of supervised learning algorithms include logistic regression (LR), support vector machine (SVM), random forest (RF), and nearest-neighbor algorithms [16,17].

Conversely, unsupervised learning does not require annotated data for training. Instead, these models utilize unlabeled data to uncover patterns that facilitate the identification of clustering or associations. Some examples of unsupervised learning include k-means, hierarchical clustering, and generative adversarial networks [18,19].

Finally, hybrid paradigms are represented by semi-supervised, weakly-supervised, and self-supervised learning approaches as a bridge between supervised and unsupervised learning. For instance, when labeled data are scarce, semi-supervised learning can integrate both labeled and unlabeled data during the training process, utilizing annotated data to guide the learning process and unlabeled data to capture additional information, thus promoting more effective generalization [20]. Weakly supervised learning models are designed to learn from partially or noisily annotated data, rather than complete and accurate labels, and make predictions or classifications based on this information [21,22]. Instead, in self-supervised learning, only unlabeled data are available, and the algorithm formulates its own learning tasks by creating surrogate tasks from the input data, such as deriving pseudolabels from the intrinsic attributes of the data [23,24].

#### Deep Learning

Traditional ML algorithms require hand-crafted features for the learning process, which is often a complex, time-consuming process. DL, a subset of ML, addresses this challenge by allowing algorithms to autonomously learn and extract pertinent features. The architecture of DL systems comprises input, hidden, and output neurons organized into multiple layers, enabling the automatic extraction of features without the need for human intervention or explicit guidance. Most DL models employed in the field of medical imaging, as discussed in this review, rely on convolutional neural networks (CNN). CNNs are specialized DL models tailored to process visual data, such as images and videos. They excel by exploiting the spatial relationships between adjacent pixels to effectively and quantitatively interpret and analyze the content. These networks comprise multiple layers of interconnected artificial neurons, referred to as convolutional layers. These layers are designed to learn filters (or kernels), which process input data and facilitate the extraction of a hierarchy of features, ranging from basic to increasingly abstract and high-level. This hierarchical feature extraction enables the network to discern not only low-level features, like edges and textures, but also high-level semantic features, such as complex structures and shapes [25]. Additionally, the architecture of the final layers of CNNs varies based on the specific tasks performed. For example, in a classification task, which could be applied in the differential diagnosis of cardiomyopathy phenotypes, the final layer comprises one or more fully connected layers followed by a softmax activation function. This configuration enables the network to generate probabilistic outputs for each class [12]—in this case, each class represents a different pathology involved in the differential diagnosis. Furthermore, multimodal neural networks can be devised to integrate visual data from various imaging modalities with other types of information, such as demographics, family history, clinical examination findings, laboratory results, and electronic health records [26]. In these networks, each data modality is processed through independent branches, each yielding a partial result. These partial results are then merged and fed into the final layers of the network to produce a comprehensive outcome. Consequently, these networks can conduct a more holistic evaluation of a patient, learning to effectively assign probabilities to different pathologies. This may lead to enhanced accuracy in medical diagnoses, as the networks consider a wide array of patient-specific data. Recently, a distinct type of neural architecture known as transformers has emerged as a popular approach, enabling the incorporation of surrounding context in the interpretation of specific portions of images or data [27]. 

## 3. Application of AI in Cardiomyopathies

Technical advancement in the use of AI within the cardiac imaging field has opened up the potential to use extensive datasets to develop models that can automatically interpret data [6,7,12,28,29]. These algorithms can support the diagnosis and treatment of patients with cardiomyopathies, allowing for early detection, outcome prediction, and prognosis evaluation [5,29,30]. AI-based algorithms have proven useful in different cardiac imaging areas, including automated image acquisition, reconstruction, and analysis [5,10,11]. In addition, AI approaches can contribute to a more efficient and standardized interpretation of coronary plaque burden, ruling out ischemic etiologies [6,11,31,32,33].

With regards to CMR, non-contrast CMR examinations combined with AI have shown promising results, enabling faster, more accessible, and cost-effective CMR images that unquestionably offer advantages in the clinical assessment of cardiomyopathy [14]. This becomes particularly relevant in light of the anticipated exponential rise in CMR examinations in accordance with the recent ESC guidelines [1].

**Figure 1:**
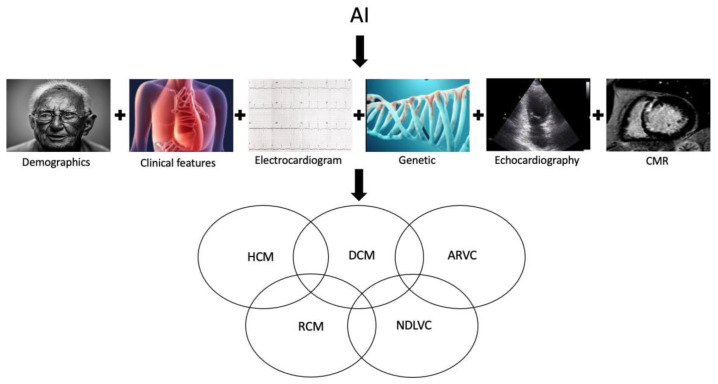
Graphical overview of an AI-based model that integrates various data, including demographic, clinical, genetic, ECG, and cardiac imaging features, to enhance the identification of a phenotype-based etiological diagnosis. AI: artificial intelligence; ARVC: arrhythmogenic right ventricular cardiomyopathy; DCM: dilated cardiomyopathy; HCM: hypertrophic cardiomyopathy; NDLVC: non-dilated left ventricular cardiomyopathy; RCM: restrictive cardiomyopathy.

AI models can merge different data, including demographic, clinical, genetic, ECG, echocardiography, CMR, and nuclear imaging parameters, allowing for a more comprehensive assessment of cardiomyopathy phenotypes (Figure 1). In recent years, radiomics features have been suggested as an additional tool to characterize features of cardiomyopathies, extracting voxel-based information from images and capturing the inherent complexity and heterogeneity of pixels in relation to their spatial “neighbors” [34,35,36,37]. Radiomics analysis generates a high number of features from each medical image, and can be coupled with AI thanks to its ability to handle a massive amount of data compared to traditional statistical analysis.

**Figure 2:**
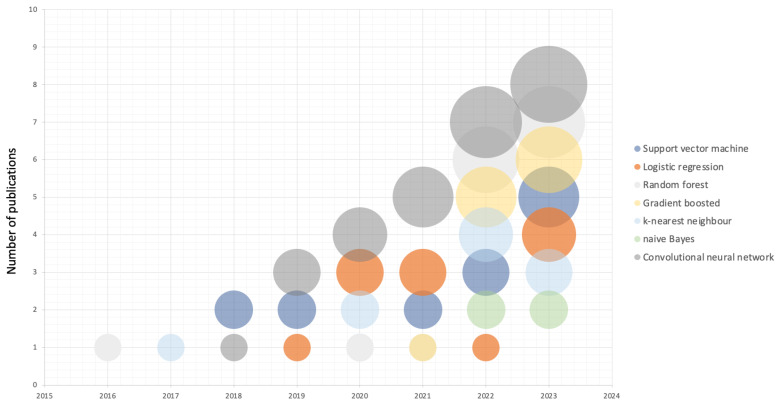
Bubble chart illustrating publications on AI-based models for the differential diagnosis of cardiomyopathy, categorized by the types of machine learning models used. Data were collected from PubMed spanning from 2016 to 2023, as of 31 December 2023. Keywords used were “cardiomyopathy”, “artificial intelligence”, “machine learning”, and “deep learning”.

AI is rising as a leading component in cardiovascular medicine, and some studies have attempted to generate AI models for the differential diagnosis of cardiomyopathy phenotypes (Figure 2). 

Table 2 summarizes previous studies regarding the application of AI models in cardiomyopathy.

Some figures were generated with an AI-based generator software, namely, Craiyon V3 (https://www.craiyon.com/).

### 3.1. Hypertrophic Cardiomyopathy

HCM is defined by the presence of elevated LV wall thickness or mass, with potential concomitant involvement of the right ventricle. Myocardial hypertrophy cannot be exclusively attributed to abnormal loading conditions (e.g., hypertension and valve disease) [1].

LV hypertrophy can arise from numerous etiologies, and the differential diagnosis of “unexplained” hypertrophy is crucial for patient management and outcomes. Indeed, some LV hypertrophy etiologies may require family screening and the implantation of a cardioverter–defibrillator [1]. AI models have demonstrated potential in classifying the underlying causes of ventricular hypertrophy, facilitating efficient diagnosis and prognosis, and preventing morbidity [38,39,42,53].

Haimovich et al. trained a CNN model using both 12-lead and single-lead electrocardiogram waveform data to differentiate between cardiac diseases associated with LV hypertrophy. The authors retrospectively analyzed 50,709 patients from the Enterprise Warehouse of Cardiology dataset, which were divided into groups characterized by cardiac amyloidosis (*n* = 304), HCM (*n* = 1056), hypertension (*n* = 20,802), aortic stenosis (*n* = 446), and other causes (*n* = 4766) [38]. The DL model based on 12-lead electrocardiograms, trained with a derivation cohort of 34,258 individuals and validated with a cohort of 16,451 individuals, achieved an AUROC of 0.95 [95% CI, 0.93–0.97] for cardiac amyloidosis, 0.92 [95% CI, 0.90–0.94] for HCM, 0.90 [95% CI, 0.88–0.92] for aortic stenosis, 0.76 [95% CI, 0.76–0.77] for hypertensive LV hypertrophy, and 0.69 [95% CI 0.68–0.71] for other LV hypertrophy etiologies. In addition, the CNN model trained on single-lead waveforms could also accurately discriminate between LV hypertrophy, with an AUROC of 0.90 [95% CI, 0.88–0.92] for cardiac amyloidosis and 0.90 [95% CI, 0.88–0.92] for HCM [38]. Similarly, an AI model based on electrocardiograms was proposed to identify pediatric patients with HCM. The CNN model was tested in 300 pediatric patients with both echocardiographic and clinical diagnoses of HCM and 18,439 healthy controls, achieving competitive diagnostic performance with an AUC of 0.98 (95% CI 0.98–0.99), sensitivity of 92%, specificity of 95%, positive predictive value of 22%, and negative predictive value of 99%. In a subgroup analysis, the DL models demonstrated a robust diagnostic performance in both sexes, as well as in genotype-positive and genotype-negative HCM patients [40].

The prediction of LV hypertrophy was also explored by an ML model that combined clinical, laboratory, and echocardiographic data. In their retrospective monocentric study, the authors evaluated 591 LV hypertrophy patients, and after splitting them into training and testing sets (75%:25%), developed three different ML models, namely, decision tree, RF, and SVM. The SVM model displayed the better diagnostic performance, with an AUROC of 0.90 (0.85–0.94), sensitivity of 0.31 (0.17–0.44), specificity of 0.96 (0.91–0.99), and accuracy of 0.80 (0.75–0.85) in the testing set.

Hwang et al. also developed a CNN long short-term memory DL algorithm to aid in the differentiation of common etiologies of LV hypertrophy (i.e., hypertensive heart disease, HCM, and cardiac amyloidosis) [41]. They achieved average AUC values of 0.962, 0.982, and 0.996 in the test set for hypertensive heart disease, HCM, and cardiac amyloidosis, respectively, and the diagnostic accuracy for the DL algorithm was significantly higher (92.3%) than for echocardiography specialists (80.0% and 80.6%) [41].

Conversely, Baeßler et al. developed a ML model using radiomics data from CMR images to detect myocardial tissue alterations in HCM. The authors retrospectively evaluated radiomics features from T1 mapping images in 32 patients with known HCM, comparing them to 30 healthy individuals. The proposed ML-based model achieved an AUC of 0.95, with a diagnostic sensitivity of 91% and a specificity of 93% [42]. 

Finally, Soto et al. developed a multimodal fusion network by integrating both electrocardiogram and echocardiogram data for the determination of the etiology of LV hypertrophy. The model was trained on more than 18,000 combined instances of examinations from 2728 patients. The proposed fusion model achieved an AUC of 0.92 (95% CI [0.862–0.965]), an F1-score of 0.73 (95% CI [0.585–0.842]), a sensitivity of 0.73 (95% CI [0.562–0.882]), and a specificity of 0.96 (95% CI [0.929–0.985]) [55].

### 3.2. Dilated Cardiomyopathy

DCM is defined as the presence of LV dilatation and systolic dysfunction that cannot solely be explained by coronary artery disease or altered loading conditions (e.g., hypertension, congenital heart disease, and valve disease) [1]. DCM is a heterogenous myocardial disease with a variable natural history and patient outcomes. The application of AI has shown the ability to define the underlying cause of LV dilatation and stratify patients according to distinct pathophysiological mechanisms [44].

The performance of an AI-based model using electrocardiogram data as a screening tool was tested by Shrivastava et al. [46]. The authors evaluated 16,471 individuals, who were divided into DCM patients (*n* = 421) and control subjects (*n* = 16,025), using a CNN model trained with 12-lead electrocardiograms. The diagnostic performance of the proposed DL models yielded an excellent diagnostic performance, with an AUC of 0.955, a sensitivity of 98.8%, a specificity of 44.8%, a negative predictive value of 100%, and a positive predictive value of 1.8% for the detection of ejection fractions ≤45% [46].

A radiomics-based ML approach to discriminate between DCM, HCM, and healthy patients was recently explored. Among the various ML models tested, RF minimum redundancy maximum relevance achieved the best performance, with an accuracy of 91.2% and average AUC values of 0.962, 0.982, and 0.996 in the test set for HCM, DCM, and healthy controls, respectively [43].

Tayal et al. proposed a ML-based approach with profile regression mixture modeling to classify patients with DCM into different sub-phenotypes. They used multiparametric data that included demographic, clinical, genetic, CMR, and proteomics parameters [44]. The proposed ML model was tested in a derivation cohort comprising 426 patients who had been prospectively enrolled in the National Institute for Health Research Royal Brompton Hospital Cardiovascular Biobank project, identifying three novel subtypes: profibrotic metabolic, mild nonfibrotic, and biventricular impairment. The DCM clusters were validated with a cohort of 239 DCM patients prospectively enrolled in the Maastricht Cardiomyopathy Registry [44]. The profibrotic metabolic cluster demonstrated the presence of mid-wall LGE in all cases (100%); 20% had diabetes, and their NYHA functional classification was predominantly class II. Conversely, among the 249 patients in the mild nonfibrotic group, none displayed myocardial fibrosis according to LGE. These patients had milder illnesses, with a significant number falling into NYHA functional class I. A separate group, consisting of 27 patients, was identified as the biventricular impairment group. These patients were notably sicker, with half of them experiencing NYHA functional class III or IV symptoms [44].

In addition, discrimination between ischemic and non-ischemic etiologies is crucial in patient management. Zhou et al. developed an ML model based on clinical and echocardiography data to discriminate between ischemic and non-ischemic LV dilatation [45]. The authors evaluated 299 patients with dilated LV, and, after splitting them into training and testing sets, developed four different ML models, namely, RF, LR, neural network, and XGBoost. The XGBoost model demonstrated the best accuracy, with a sensitivity of 72%, specificity of 78%, accuracy of 75%, F-score of 0.73, and AUC of 0.934. In addition, the ML model demonstrated good accuracy in terms of discriminating between different etiologies in an external validation cohort, with a sensitivity of 64%, a specificity of 93%, and an AUC of 0.804 [45].

### 3.3. Non-Dilated Left Ventricular Cardiomyopathy and Arrhythmogenic Right Ventricular Cardiomyopathy

ARVC is characterized by the presence of right ventricle dilatation and/or dysfunction, along with histological involvement and/or electrocardiographic abnormalities, according to the Task Force criteria [1,56]. 

Conversely, NDLVC is a newly proposed category of cardiomyopathy. It is defined by the presence or absence of left ventricular systolic impairment, either regionally or globally, along with non-ischemic myocardial scarring or the presence of fatty replacement [1]. This includes a heterogenous group of patients previous described as having dilated cardiomyopathy without dilated LV, arrhythmogenic left ventricular cardiomyopathy, left-dominant arrhythmogenic cardiomyopathy, or arrhythmogenic dilated cardiomyopathy. These patients do not fulfill the Task Force criteria [1].

AI-based models promise to facilitate the diagnosis of these cardiomyopathy subtypes [47,49].

Zhang et al. developed an algorithm for distinguishing between arrhythmogenic cardiomyopathy and dilated cardiomyopathy based on ML algorithms from two public datasets containing myocardial samples. Several ML models were tested, including SVM, naive Bayes, decision tree, K147 nearest neighbor, gradient boosting machine, extreme gradient boosting, and RF, to identify key candidate genes with which to construct a predictive model for favoring separation between arrhythmogenic cardiomyopathy and dilated cardiomyopathy. The RF model achieved the best performance, with an AUC of 0.86 [47]. Similarly, Bleijendaal et al. developed ML and DL models based on typical electrocardiographic features to diagnose Phospholamban p.Arg14del cardiomyopathy, a well-known mutation associated with the development of dilated and/or arrhythmogenic cardiomyopathy [48]. The authors compared the proposed AI models with an expert cardiologist, demonstrating that the AI models outperformed the expert cardiologist in term of accuracy and sensitivity in both the training and testing datasets. In addition, T-wave morphology was identified as the most important electrocardiographic feature used to classify PLN p.Arg14del carriers [48].

Another ML model based on electrocardiograms was recently developed by Papageorgiou et al. to discriminate patients with ARVC. The authors proposed the utilization of a CNN for the detection of arrhythmogenic heart disease, achieving 99.98% accuracy, 99.96% specificity, and 99.98% sensitivity during the training phase and 98.6% accuracy, 98.25% specificity, and 98.9% sensitivity when tested [49].

### 3.4. Restrictive Cardiomyopathy

RCM is defined by the presence of non-dilated left or right restrictive pathophysiology, with normal ventricular wall thickness and normal or reduced diastolic and/or systolic volume in one or both ventricles [1]. The distinction between RCM and constrictive pericarditis could be challenging because of the overlap in clinical presentation and restrictive flow patterns with diastolic dysfunction [57]. Chao et al., by using an apical four-chamber view from transthoracic echocardiography studies, trained a ResNet50 deep learning model, achieving a performance with an area under the curve of 0.97, to differentiate constrictive pericarditis from cardiac amyloidosis [50].

Sengupta et al. designed a ML approach that combines clinical parameters, conventional echocardiographic, and speckle tracking echocardiography data to discriminate between RCM and constrictive pericarditis [51]. The associative memory classifier demonstrated the best diagnostic performance, with an accuracy of 93.7% and an AUC of 96.2% [51]. 

Another cardiac condition that can result in a restrictive pattern is thalassemia. AI-based models have been proposed to identify and measure iron deposits in the heart, enabling early recognition of myocardial dysfunction and subsequent prompt treatment. Taleie et al. proposed a ML model to discriminate between beta-thalassemia major patients with myocardial iron overload from those without myocardial iron overload based on radiomic features extracted from echocardiography images. Among the various ML models tested, maximum relevance–minimum redundancy–extreme gradient boosting achieved the best performance, with an AUC of 0.73, accuracy of 0.73, specificity of 0.73, and sensibility of 0.73 [37]. Similarly, a ML model to evaluate heart iron overload was proposed by Asmarian et al. achieving an AUC of 0.68 (95% CI 0.60, 0.75) [52].

Among RCM, Fabry diseases should be taken into account. Due to the heterogenous and multisystemic clinical presentation, this diagnosis could be challenging. An AI-based approach was recently proposed to identify patients affected by Fabry diseases using health record data from 4978 patients with Fabry diseases and 1,000,000 healthy controls. The AI model was trained using a training cohort comprising 75% of all patients, who were selected at random to construct a statistical “phenotypic biomarker” for Fabry disease. Then, the authors predicted the likelihood of having Fabry disease for each patient in the testing cohort, which included 25% of all patients. This method achieved a strong analytic performance, with an AUC of 0.82 [58].

Eckstein et al. developed a supervised ML model that integrates various strain parameters from the right atrium, left atrium, and right ventricle, as well as cardiac function metrics, to identify cardiac amyloidosis using multiple machine learning classifier algorithms, including k-nearest neighbor, linear and non-linear SVM, and decision trees. The non-linear SVM exhibited the best performance, achieving an accuracy rate of 90.9% and an AUC of 0.996, outperforming other machine learning algorithms in their study [53].

### 3.5. Other Cardiac Syndromes Associated with Cardiomyopathy Phenotypes

In accordance with the recent ESC guidelines, this category encompasses LV hypertrabeculation and Takotsubo syndrome [1]. The term “LV hypertrabeculation” is recommended over LV non-compaction, especially if this phenomena is transient or if it clearly occurs during adulthood [1]. Takotsubo syndrome is defined as a peculiar pattern of LV dysfunction that presents, in its most typical variant, as apical ballooning and hyperkinesis of LV basal segments in the absence of obstructive coronary disease [1,59,60,61]. AI models have been applied to identify these cardiac syndromes associated with cardiomyopathy phenotypes [36,54].

Cau et al. designed an ML model that combines CMR parameters and demographics factors to identify patients with Takotsubo syndrome among individuals with acute chest pain. The authors retrospectively enrolled three groups of patients: those with Takotsubo cardiomyopathy, patients with acute myocarditis, and healthy individuals. To assess the model’s performance, they employed five different tree-based ensemble learning algorithms, specifically RF, extremely randomized Trees, bagging of trees, adaptive boosting, and extreme gradient boosting. The extremely randomized trees ML algorithm showcased a sensitivity of 92% (with a 95% confidence interval of 78–100), a specificity of 86% (95% CI: 80–92), and an AUC of 0.94 (95% CI: 0.90–0.99) in the diagnosis of Takotsubo syndrome. Furthermore, the proposed model outperformed clinical reader diagnoses, exhibiting an average increase in AUC of 0.42 (representing an 80% improvement), an increase in sensitivity of 0.08 (equivalent to a 10% boost), and an increase in specificity of 0.618 (indicating a 257% enhancement). Importantly, this improved diagnostic performance was achieved with a significantly shorter duration of analysis, taking only 0.26 s compared to 560 s for the clinical reader [54].

An ML approach based on radiomics data was developed to automatically differentiating LV non-compaction from other cardiomyopathy phenotypes, including HCM and DCM [36]. The authors extracted a set of 420 radiomics features from 118 patients (including 37 DCM, 25 HCM, and 35 left ventricle non-compaction patients, as well as 21 healthy control individuals). The radiomics model to discriminate cardiomyopathy phenotypes was built with different consecutive steps, namely, (1) removing radiomics features that were highly correlated; (2) normalization to introduce the features into ML models; (3) feature selection, selecting the best features based on univariate statistical tests; and (4) training and testing different ML models, such as SVM, multi-class RF, and multi-class LR, for classification. The radiomics models for the automated diagnosis of left ventricle non-compaction achieved excellent diagnostic performance, with AUC values of 0.95 [36].

## 4. Limitations and Future Directions

AI has the potential to reduce costs and save physicians’ time by analyzing complex data and enabling phenotype-based etiological diagnosis, but several challenges must be addressed for its effective implementation in real-world clinical practices. These challenges include the heterogeneity of collected data, issues related to reproducibility, the necessity of internal and external validation, interpretability concerns, and ethical and legal considerations.

### 4.1. Data Heterogeneity and Reproducibility

First, building robust AI algorithms demands a significant amount of data, along with complex software and infrastructure. To ensure the reliability and generability of AI models, an ample supply of annotated data from different centers is essential. It is also crucial to homogenize data in terms of their formats, definitions, and quality control protocols, as well as standardize data acquisition across different imaging modalities. Indeed, insufficient data can lead to overfitting or underfitting, resulting in inaccurate predictions. As the array of AI algorithms continues to expand, there is a pressing need for research standardization to ensure the reproducibility of the proposed models. Reproducibility, in this context, refers to the ability to duplicate prior results using the same methods employed in the original work. Achieving technical reproducibility in AI studies is contingent on the release of both the code and dataset by the study group [62,63,64]. For this reason, open-access datasets and algorithm codes are foundational to advancing the field of AI in healthcare by providing the necessary resources for training, external validation, and reproducibility, as well as mirroring real-world data. Table 2 summarizes the public datasets and available code discussed in the current review. 

### 4.2. Ethical and Legal Issues

Second, ethical and legal concerns regarding the implementation of AI in healthcare encompass the use of patient data for the development of AI-based models. The generation of large and homogeneous datasets gives rise to significant issues, particularly in the domains of data privacy and security. A notable area of apprehension pertains to informed consent, surveillance, and the violation of individuals’ data protection rights. This concern extends to the possibility of undisclosed uses of the data by entities distinct from the individual from whom the data were obtained [65,66]. Moreover, the vulnerability of information collected for and by AI systems to hacking attempts introduces an additional layer of worry. Instances of unauthorized access and data breaches pose threats to the integrity and confidentiality of sensitive medical information [67,68]. Privacy and data protection laws have taken proactive steps to address these concerns. These efforts aim to establish frameworks ensuring transparency, safety, and ethical standards in the handling, processing, and sharing of data related to healthcare [69,70,71]. Ensuring the fairness of AI models is a critical concern, particularly due to the potential for biases to be embedded in these models, especially when training data exhibit imbalances. This imbalance can lead to significant variations in the performance of AI models across demographic groups, such as sex and race, potentially worsening existing healthcare disparities. A comprehensive examination of fairness and equity in the development and deployment of AI in healthcare is essential. Addressing these challenges is vital for unlocking the full potential of AI while safeguarding privacy, promoting transparency, and ensuring equitable healthcare outcomes for all [72,73,74,75].

### 4.3. Model Transparency, Explainability, and Validation

Third, some AI-generated models, especially those based on DL, are considered “black box” systems that do not offer any insight or explanations about how the output is obtained, limiting clinical applicability due to their lack of transparency and interpretability. To overcome the significant challenges presented by “black box” models, recent studies have focused on developing AI models that are both explainable and interpretable [12,76,77]. Specifically, interpretability refers to the human ability to understand cause and effect within an AI model. Conversely, explainability refers to the capability to elucidate an AI-generated decision-making process using language that is intelligible to humans, enabling an understanding of why a model produces a specific outcome [12,76,77]. Many ML models are designed to be intrinsically interpretable (e.g., LR, linear regression, decision trees, K-nearest neighbors) due to their simple structure and inherent logical relationships to their components [78,79]. In models of greater complexity, the direct interpretation process becomes intricate and rapidly extends beyond quick logical comprehension, requiring post-modeling explainability [80]. Additionally, an interpretable model should undergo clinical validation through an evaluation of whether the interpretations provided by the model are as effective enough to support healthcare decision making as expert advice [81].

Moreover, when a dataset is available, internal [82,83,84] and external validation of the model, as well as studies conducted in real-world settings, are also crucial to assessing the practical utility of deployed models [85,86]. Indeed, a significant barrier to the widespread adoption of AI algorithms is the lack of an evaluation of their clinical impacts through prospective studies, leaving the benefits of AI approaches largely theoretical. Similarly, there are no studies assessing the economic or health system advantages associated with population-level approaches to cardiomyopathy using AI algorithms. 

### 4.4. Future Directions

Randomized trials are crucial for assessing the application of AI models to the differential diagnosis of cardiomyopathy phenotypes across different centers, minimizing bias through sex and minority representation. To enhance the applicability of these models, it is mandatory not only to collect extensive, homogenous datasets with external validation, quality controls, and explainable and interpretable approaches, but also to inform physicians about the usefulness of these models. Additionally, developing a privacy protection algorithm with encryption and AI techniques is necessary for secure and generalizable models.

AI models used in cardiomyopathy-oriented approaches with multiparametric data may represent a potential advancement in cardiomyopathy management. This includes disease classification, early diagnosis, risk stratification, and early detection of complications, as well as prognosis and tailored treatment. Additionally, AI imaging pre-processing and post-processing approaches are undoubtedly useful in clinical practice to optimize clinical workflows and reduce the cost of cardiovascular healthcare, providing a solution to the exponential increase in cardiovascular examinations [28,87,88]. AI algorithms exhibit the capacity to manage larger and more intricate datasets in comparison with traditional medical models, and do not require the pre-specification of predictors or interactions, thereby facilitating the identification of novel relationships [89,90]. These AI-based models allow us to bridge the gap between the pathogenesis of diseases, genotypes, and phenotypes of cardiomyopathies, enabling personalized medicine not only for the individual, but for the entire family.

## 5. Conclusions

The implementation of AI-based models for the differential diagnosis of cardiomyopathy phenotypes holds promise in terms of significantly enhancing daily clinical practice in cardiovascular healthcare. The multiparametric approach to evaluating and discriminating patients with suspected cardiomyopathy has been demonstrated to be crucial in identifying the cardiomyopathy phenotypes and the underlying etiological diagnoses. AI models have the potential to create composite algorithms that include demographics and laboratory data, clinical parameters, molecular, and cardiac imaging features to automate the detection and diagnosis of cardiomyopathies. Despite the potential advantages of these AI algorithms in cardiovascular healthcare, their applicability is currently limited due to several challenges. These concerns include the lack of homogenous datasets with external validation and real-world evaluations, the limited interpretability and robustness of the models, and lingering issues regarding trust in AI-generated approaches. To expedite the clinical implementation of these models as valuable tools in cardiomyopathy diagnosis, it is crucial to design future multicenter trials with homogenous and open-access datasets to achieve universal applicability of these AI models.

## Figures and Tables

**Table 1:** An overview of AI models with brief descriptions.

Algorithm	Overview	Advantages	Disavantages
Support vector machine	A supervised ML model used for both classification and regression. It works by finding an hyperplane that maximally separates data points of different classes in a high-dimensional space, aiming to maximize the margin between the classes.	Effectively handles unstructured and semi-structured data Low generalization error	Requires long training time for large datasets Results may be difficult to interpret
Logistic regression	A supervised ML algorithm used for binary classification. It models the probability of an instance belonging to a particular class using the logistic function, and the decision boundary is a linear combination of input features.	Provides insight into feature relevance Efficient for small datasets Rapid training	Assumption of linearity Sensitivity to outliers
Random forest	A supervised ML algorithm used for classification and regression tasks. It builds multiple decision trees during training and merges their predictions to improve accuracy and robustness. Each tree is trained on a random subset of the data, and the final prediction is determined by a majority vote (for classification) or an average (for regression).	High accuracy in capturing complex relationships in data Efficient on large datasets Provides insight about feature relevance	Sensitive to small changes in data Bias towards dominant classes
Gradient-boosted decision tree	An ensemble learning technique used for both classification and regression. It builds a series of decision trees sequentially, with each tree correcting the errors of the previous ones. It combines the predictions of individual trees to create a strong predictive model.	High accuracy in capturing complex relationships in data Provides insight into feature relevance Effective on structured and unstructured data	Requires long training time for large datasets Complex interpretability
k-nearest neighbour	A supervised ML algorithm for classification and regression that predicts a data point’s label or value based on the majority of its nearby neighbors in the dataset. The “k” represents the number of neighbors considered for the prediction.	No training period Supports dynamic data addition Efficient for small datasets	Poor performance with large datasets Impact of irrelevant features Sensitivity to outliers and missing data
Naive Bayes	A supervised ML algorithm for classification. It is based on Bayes’ theorem and assumes independence between features. The algorithm calculates the probability of a data point belonging to a particular class by considering the probabilities of its individual features.	Simple and fast Requires small amount of training data Less sensitive to irrelevant features	Assumption of feature independence “Zero Probability” issue Sensitivity to outliers and missing data
Convolutional neural network	A DL algorithm designed for image and video recognition. It uses convolutional layers to automatically and adaptively learn spatial hierarchies of features from input data.	Learns hierarchical features from spatial data Allows parameter sharing, reducing overfitting Automated feature learning	Large datasets needed Requires long training time Complex interpretability

**Table 2:** Previous studies regarding the application of AI models in cardiomyopathy. ARVC: arrhythmogenic right ventricular cardiomyopathy; CCN: convolutional neural network; DCM: dilated cardiomyopathy; DT: decision tree; HCM: hypertrophic cardiomyopathy; LR: logistic regression; K-NN: K-nearest neighbor; NDLVC: non-dilated left ventricular cardiomyopathy; RCM: restrictive cardiomyopathy; RF: random forest; SVM: support vector machine; TTE: transthoracic echocardiogram; XGboost: extreme gradient boosting.

Authors	Year	Patients	Cardiomyopathy Phenotypes	Variables	AI Models	Programming Languages	Model Interpretability	Validation	Public Datasets and Code	Results
Haimovich et al. [38]	2023	50,709	HCM	ECG data	CNN	Python v3.8 (Python Software Foundation, Beaverton, Oregon)	Black box	External validation	No public datasets No algorithm code available	The DL model achieved an AUROC of 0.95 [95% CI, 0.93–0.97] for cardiac amyloidosis, 0.92 [95% CI, 0.90–0.94] for hypertrophic cardiomyopathy, 0.90 [95% CI, 0.88–0.92] for aortic stenosis, 0.76 [95% CI, 0.76–0.77] for hypertensive left ventricle hypertrophy, and 0.69 [95% CI 0.68–0.71] for other left ventricle hypertrophy etiologies.
Beneyto et al. [39].	2023	591	HCM	Clinical, laboratory and TTE data	DT, RF, and SVM	R packages version 4.1.1 (R Foundation for Statistical Computing, Vienna, Austria)	Inherently interpretable model Post-modeling explainability	Internal validation	No public datasets No algorithm code available	The proposed ML model achieved an AUROC of 0.90 (0.85–0.94), sensitivity of 0.31 (0.17–0.44), specificity of 0.96 (0.91–0.99), and accuracy of 0.80 (0.75–0.85) in the testing set.
Siontis et al. [40]	2021	18,739	HCM	ECG data	CNN	Python (Python Software Foundation, Beaverton, Oregon)	Black box	External validation	No public datasets No algorithm code available	The DL model achieved an AUC of 0.98 (95% CI 0.98–0.99), sensitivity of 92%, specificity of 95%, positive predictive value of 22%, and negative predictive value of 99%.
Hwang et al. [41]	2022	930	HCM	TTE data	CNN	R packages version 4.1.1 (R Foundation for Statistical Computing, Vienna, Austria)	Black box	Internal validation	No public datasets Algorithm code available (https://github.com/djchoi1742/Echo_LVH) accessed on 30 December 2023	The DL model achieved average AUCs of 0.962, 0.982, and 0.996 in the test sets for hypertensive heart disease, HCM, and cardiac amyloidosis, respectively.
Baeßler et al. [42]	2018	32	HCM	Radiomics features	Machine learning	R packages Version 3.4.0 (R Foundation for Statistical Computing, Vienna, Austria)	Post-modeling explainability	Internal validation	No public datasets No algorithm code available	The proposed ML-based model achieved an AUC of 0.95, with a diagnostic sensitivity of 91% and a specificity of 93%.
Zhang et al. [43]	2023	238	HCM, DCM	Radiomics features	Multilayer perceptron, DT, RF, LR, XGboost, SVM, naive Bayes, K-nearest neighbor, and ensemble learning	Python version 4.1.1 (Python Software Foundation, Beaverton, Oregon)	Inherently interpretable model Post-modeling explainability	Internal validation	Public datasets (https://acdc.creatis.insalyon.fr/description/databases.html; https://www.ub.edu/mnms/) accessed on 30 December 2023 No algorithm code available	The proposed ML model achieved an accuracy of 91.2% and average AUCs of 0.962, 0.982, and 0.996 in the test sets for HCM, DCM, and healthy controls, respectively.
Tayal et al. [44]	2022	665	DCM	Demographic, clinical, genetic, CMR, and proteomics parameters	RF	Premium R packages (R Foundation for Statistical Computing, Vienna, Austria)	Post-modeling explainability	External validation	No public datasets No algorithm code available	The proposed ML model identified three novel DCM subtypes: profibrotic metabolic, mild nonfibrotic, and biventricular impairment.
Zhou et al. [45]	2023	399	DCM	Clinical and TTE data	RF, LR, neural network, and XGBoost	R packages version 3.6.2 (R Foundation for Statistical Computing, Vienna, Austria)	Inherently interpretable model Post-modeling explainability	External validation	No public datasets No algorithm code available	The ML model achieved good accuracy in discriminating between different etiologies in an external validation cohort with a sensitivity of 64%, a specificity of 93%, and AUC of 0.804.
Shrivastava et al. [46]	2021	16,471	DCM	ECG data	CNN	Python version 3.9 (Python Software Foundation, Beaverton, Oregon)	Black box	Internal validation	No public datasets No algorithm code available	The diagnostic performance of the proposed DL models yielded an AUC of 0.955, a sensitivity of 98.8%, and specificity of 44.8%, a negative predictive value of 100%, and a positive predictive value of 1,8%
Zhang et al. [47]	2022	57	ARVC/NDLVC	Transcriptome profiles from human hearts	SVM, naive Bayes, DT, K-NN, gradient-boosting machine, XGboost, and RF	R packages version 4.1.3 (R Foundation for Statistical Computing, Vienna, Austria)	Inherently interpretable model Post-modeling explainability	External validation	Public datasets (http://www.ncbi.nlm.nih.gov/geo; https://www.mdpi.com/2073-4425/11/12/1430/s1) accessed on 30 December 2023 No algorithm code available	Random forest achieved the best performance, with an AUC of 0.86 in discriminating between arrhythmogenic cardiomyopathy and dilated cardiomyopathy.
Bleijendaal et al. [48]	2020	310	ARVC/NDLVC	ECG data	CNN, long short-term memory, K-NN, LR, multilayer perceptron, RF, SVM, XGboost	Python version 3.9 (R Foundation for Statistical Computing, Vienna, Austria)	Inherently interpretable model Post-modeling explainability	External validation	No public datasets Algorithm code available (https://github.com/L-Ramos/CardiologyAI.) accessed on 30 December 2023	The proposed ML and DL models outperformed expert cardiologists in terms of accuracy and sensitivity.
Papageorgiou et al. [49]	2022	183	ARVC/NDLVC	ECG data	CNN	R packages version 4.1.0 (R Foundation for Statistical Computing, Vienna, Austria)	Post-modeling explainability	Internal validation	No public datasets No algorithm code available	The CNN model achieved 99.98% accuracy, 99.96% specificity, and 99.98% sensitivity during the training phase and 98.6% accuracy, 98.25% specificity, and 98.9% sensitivity when tested.
Chao et al. [50]	2023	381	RCM	TTE data	CNN	Python version 4.1.1 (Python Software Foundation, Beaverton, Oregon)	Post-modeling explainability	External validation	No public datasets No algorithm code available	The DL model yielded an AUC of 0.97 in differentiating constrictive pericarditis vs. cardiac amyloidosis.
Sengupta et al. [51]	2016	94	RCM	Clinical parameters, conventional TTE, and speckle tracking TTE data	Associative memory classifier, RF, K-NN, and SVM	R packages version 3.3 (R Foundation for Statistical Computing, Vienna, Austria)	Post-modeling explainability	Internal validation	No public datasets No algorithm code available	The proposed ML approach achieved an accuracy of 93.7% and an AUC of 96.2%.
Taleie et al. [37]	2023	91	RCM	radiomic features	K-NN, LR, multi-layer perceptron, RF, SVM, and XGboost	R packages version 4.0 (R Foundation for Statistical Computing, Vienna, Austria)	Inherently interpretable model Post-modeling explainability	Internal validation	No public datasets No algorithm code available	The ML model achieved the best performance, with an AUC of 0.73, accuracy of 0.73, specificity of 0.73, and sensibility of 0.73.
Asmarian et al. [52]	2022	624	RCM	Clinical and laboratory data	RF, gradient boost model, and LR.	R packages version 4.1.0 (R Foundation for Statistical Computing, Vienna, Austria)	Inherently interpretable model Post-modeling explainability	Internal validation	No public datasets No algorithm code available	The ML model yielded an AUC of 0.68 in evaluating heart iron overload.
Eckstein et al. [53]	2022	96	RCM	CMR strain and function parameters	k-NN, SVM, and DT	Python Version 3.8.12 (Python Software Foundation, Beaverton, Oregon)	Inherently interpretable model Post-modeling explainability	Internal validation	No public datasets No algorithm code available	The ML-based model achieved an accuracy rate of 90.9% and an AUC of 0.996.
Cau et al. [54]	2022	43	Takotsubo syndrome	CMR parameters, demographics data	RF, bagging of trees, adaptive boosting, and XGboost	R packages version 4.1.0 Python version 3.9 (Python Software Foundation, Beaverton, Oregon)	Inherently interpretable model Post-modeling explainability	Internal validation	No public datasets No algorithm code available	The extremely randomized trees ML algorithm showcased a sensitivity of 92% (with a 95% confidence interval of 78–100), a specificity of 86% (95% CI 80–92), and an AUC of 0.94 (95% CI 0.90–0.99) in the diagnosis of Takotsubo syndrome.
Izquierdo et al. [36]	2021	118	Left ventricle non-compaction	Radiomics features	SVM, RF, LR	Python version 3.7.9 (Python Software Foundation, Beaverton, Oregon)	Inherently interpretable model Post-modeling explainability	Internal validation	No public datasets No algorithm code available	The radiomics models for the automated diagnosis of left ventricle non-compaction achieved excellent diagnostic performance, with AUC values of 0.95.

## Data Availability

No new data were created or analyzed in this study. Data sharing is not applicable to this article.
